# Editorial: Metabolism, gut microbiome, and cancer

**DOI:** 10.3389/fonc.2025.1647690

**Published:** 2025-07-18

**Authors:** Yuanyuan Li, Trygve O. Tollefsbol

**Affiliations:** ^1^ Department of Nutrition and Food Science, College Park, MD, United States; ^2^ Department of Biology, University of Alabama at Birmingham, Birmingham, AL, United States

**Keywords:** gut microbiome, metabolism, metabolites, cancer, immune, targeted therapy, prevention, tumorigenesis

Aberrant metabolic alterations are fundamental drivers for tumorigenesis. Targeting cancer metabolism can provide novel diagnostic biomarkers and intervention targets for cancer research. The gut microbiome has been increasingly recognized as a critical regulator of host health and disease. Accumulating evidence implicates that the gut microbiome plays major roles in cancer development and progression, as well as influencing individual responses to anticancer therapies through modulation of immune responses and metabolic pathways. Gut microbiota-derived metabolites can also act as key modulators connecting the gut microbiome status to cancer development and treatment outcomes. Therapeutic interventions targeting the gut microbiota or its metabolites have demonstrated fundamental potential for anticancer drug development. Therefore, the interplay between metabolism, the gut microbiome, and cancer has emerged as a pivotal area of cancer research, offering novel insights into tumorigenesis and potential therapeutic avenues.

This Research Topic entitled “*Metabolism, Gut Microbiome, and Cancer*” in *Frontiers in Oncology* has collected ten scholarly articles that reflect the latest advances in this interdisciplinary field. Collectively, this Research Topic provides new insights into the complex interrelationships between host metabolism and the gut microbiome in cancer biology. It also highlights promising targets for future therapeutic interventions aimed at modulating microbial and metabolic pathways to improve cancer prevention and treatment.

The gut microbiome affects the efficacy and treatment outcomes of cancer chemotherapy. Ginwala et al. reported an original study investigating how the urinary microbiome influences bladder cancer treatment outcomes. Researchers have identified the enriched presence of certain bacterial species, such as *Granulicatella* and *Proteus*, in patients who did not respond to neoadjuvant chemotherapy, while *E. faecalis* was more prevalent in the responders, suggesting that specific microbial profiles may affect chemotherapeutic efficacy. Additionally, this study revealed that some bacteria within the urinary microbiome can metabolize gemcitabine, a common chemotherapy agent for bladder cancer, potentially reducing its effectiveness. This finding highlights the importance of individualized microbial compositions that help develop personalized treatment strategies for bladder cancer patients.

Metabolic reprogramming is considered as a hallmark of cancer progression and metastasis featured by abnormalities in glucose, glutamine and lipid metabolism. In a review article, Lu et al. emphasized the alterations in glucose, lipid and amino acid metabolism that may drive primary liver cancer. Better understanding of these metabolic alterations offers insights into potential therapeutic strategies targeting metabolic pathways to improve liver cancer treatment outcomes. Epigenetic regulations including histone modification have been shown to affect immune regulation, metabolic reprogramming, and tumor growth. Another review article by Al-Malsi et al. in this Research Topic examined how histone lactylation could serve as a novel epigenetic biomarker linking metabolic reprogramming to gene expression changes in cancer diagnosis and treatment. These insights open avenues for targeted therapies aimed at modulating epigenetic pathways to improve cancer treatment outcomes.

The interplay between the gut microbiome, host metabolism, and immune responses plays a critical role in cancer risk and therapy outcomes, representing a key element in cancer pathophysiology. Several studies in this Research Topic have utilized Mendelian randomization to identify causal relationships between gut microbiota, metabolites, immune biomarkers and cancer risk in specific populations. For example, Wang et al. studied the causal relationship between the gut microbiota, metabolites, cytokines, and prostate cancer risk in East Asian populations. Pan et al. explored the genetic underpinnings linking serum metabolites to prostate cancer risk through analyzing existing databases such as metabolome-based genome-wide association study (GWAS). This research identified three metabolites including fructose, N1-methyl-3-pyridone-4-carboxamide, and 12-hydroxyeicosatetraenoate as genetically associated with increased prostate cancer risk. A study conducted by Huang et al. focused on determining how genetically predicted basal metabolic rate can influence the risk of benign neoplasms in bone and cartilage through analyzing the single nucleotide polymorphisms in the European population. In addition, Huang and Zheng conducted a bidirectional Mendelian randomization analysis and revealed that certain metabolic markers such as higher total bilirubin, urate, and serum calcium are potentially inversely related to specific lung cancer subtypes (e.g., small cell and non-small cell), while elevated alkaline phosphatase may be associated to an increased risk of lung adenocarcinoma. Collectively, leveraging large datasets, these studies reveal a complex interplay between gut microbial alterations, metabolic pathways, and cancer outcomes, highlighting the potential influence of host-microbiome interactions. A deeper understanding of these relationships may inform the development of predictive biomarkers and novel therapeutic targets in cancer.

This Research Topic also covers cross-disciplinary areas in cancer research such as drug development. By targeting vulnerabilities in DNA damage repair pathways, synthetic lethality offers a powerful approach in anticancer drug development, enabling more precise and personalized treatment options. The review article by Gong et al. discussed the application of synthetic lethality strategies on enhancing therapeutic safety by considering metabolic contexts. Additionally, a case study reported by Huang el al. presented a rare instance of hepatocellular carcinoma with isolated pelvic metastasis, highlighting the importance of monitoring alpha-fetoprotein during post-surgery care.

Lastly, a perspective article by Nair et al. discussed the importance of viewing cancer as a metabolic disease. The authors provide an overview of evidence-based strategies that support the use of integrative medicine in targeting cancer metabolism. They emphasize the importance of uniting genetic and metabolic theories of cancer to enhance treatment effectiveness and patient care. The integrated approach underscores the importance of personalized medicine that tailors the individual’s unique genetic, metabolic, and psychosocial profile, leading to improved treatment and survival outcomes.

In summary, the ten articles in this Research Topic highlight the multifaceted roles of the gut microbiome and metabolome in cancer biology. They emphasize the potential of targeting microbial communities and metabolomic outputs as part of integrated cancer prevention and treatment strategies. Importantly, these findings underscore a growing recognition that cancer is not solely a genetic disease but one deeply influenced by complicated interactions between host microbes and metabolic environment. As such, incorporating microbiome and metabolomic profiling into precision oncology could lead to more personalized, responsive interventions. Continued interdisciplinary research that bridges oncology, microbiology, systems biology, and metabolomics holds great promise for unveiling novel mechanisms of tumorigenesis and unlocking new therapeutic and diagnostic pathways for cancer patients.

